# American Asylums for the Insane
*1. The Pennsylvania Hospital for the Insane for the year 1861.2. The Friends' Asylum, for the fiscal year 1861–62.3. The Western Pennsylvania Hospital, for the year 1861.4. The Bloomingdale Asylum, for the year 1861.5. The McLean Asylum, for the year 1861.6. The Massachusetts State Hospital at Northampton, for the fiscal year 1860–61.7. The Longview Asylum, for the year 1861.8. The New Hampshire Asylum, for the fiscal year 1861–62.


**Published:** 1863-04

**Authors:** 


### Art. VIII.—AMERICAN ASYLUMS FOR THE INSANE.*

1. Should the progress of the next century, in the province of psychiatry, be equal to that of the century at the termination of which we write, the problem of The Perfect Hospital for the Insane can hardly fail of being solved. In this remark we allude not alone to the minor details of organization, classification, discipline, and the diversified means for the administration of a perfect moral treatment, but to the great primary question of the character of the hospital as it regards the sexes, and the duration of the disease of its patients. The Germans have tried almost every form of hospital in these respects, without, hitherto, arriving at unanimity of opinion upon the subject; but, so far as our knowledge extends, they have *not* tried that which has recently been adopted at the *Pennsylvania Hospital for the Insane*—the intermingling of both recent and chronic cases, but the separation of the sexes by buildings somewhat remote each from the other, yet both under the same general administration and the same physician in chief. It is an experiment challenging the attention and the interest not only of American but also of European colonists. The testimony borne by Dr. Kirkbride in relation to the subject, after his second year of trial, is as follows:—

"Another year's experience has confirmed our previous impressions of the great value of the new arrangements, and has demonstrated more fully, if additional proof were still wanting, that the treatment of the two sexes in different buildings, as here conducted, without having, as far as we can discover, a

---

* 1. The Pennsylvania Hospital for the Insane for the year 1861.
2. The Friends' Asylum, for the fiscal year 1861–62.
3. The Western Pennsylvania Hospital, for the year 1861.
4. The Bloomingdale Asylum, for the year 1861.
5. The McLean Asylum, for the year 1861.
6. The Massachusetts State Hospital at Northampton, for the fiscal year 1860–61.
7. The Longview Asylum, for the year 1861.
8. The New Hampshire Asylum, for the fiscal year 1861–62.

single objection, has unquestionably many and important advantages."

This certainly looks encouraging; but it is no more so than we expected after having visited the Institution, examined both departments with all their admirable arrangements and appliances, and duly considered the whole establishment in its relation and adaptation to the wants of the insane.

| | Men. | Women. | Total. |
|---|---|---|---|
| Patients in hospital Jan. 1st, 1861 . | 151 | ... 123 | ... 274 |
| Admitted in course of the year . . . | 96 | ... 86 | ... 182 |
| Whole number . . . . . . . . . | 247 | ... 209 | ... 456 |
| Discharged, including deaths . . . | 119 | ... 82 | ... 201 |
| Remaining, Dec. 31st, 1861 . . . . | 128 | ... 127 | ... 255 |
| Of the discharged, there were cured . | 46 | ... 46 | ... 92 |
| Died . . . . . . . . . . . . | 21 | ... 8 | ... 29 |

*Causes of Death.*—"Acute maniacal disease," 10; chronic softening of the brain, 5; pulmonary consumption, 4; exhaustion of chronic mania, 3; apoplexy, 3; suicide, 1; disease of heart, 1; dropsy, 1; "effects of a long journey," 1.

"The sixteenth annual course of lectures and evening entertainments, which terminated at the usual period of the last summer, was, as those heretofore given, of nine months' duration—three times a week at least, at each department—and occupying about two hundred and fifty evenings. These entertainments being on alternate evenings at the two buildings, anything of special interest occurring at one, can be enjoyed by patients from the other; and this has been frequently done. A company of forty ladies has on several occasions gone to the new building during the past year.

"Every year adds to the conviction of the great importance of these entertainments in the management of a hospital for the insane. It is quite possible, with proper zeal and determination, to make the evening hours in such institutions the most pleasant in the whole day. Without some decided effort, however, this period is apt to become specially tiresome, and the wards, then, to present their most listless and discouraging appearance. From sunset to bedtime there should be a persistent effort on the part of all, to have something on hand that will, at least to some extent, excite the interest and attract the attention of even those of least mental activity. The first step is to have the corridors and parlours cheerfully lighted and comfortably furnished, to have in progress agreeable work, pleasing games, interesting to lookers-on as well as players, pictures of various kinds, pleasant reading or music, and varying novelties that those best qualified for positions here will be constantly suggesting. In this work the officers of course must take the lead. There must be nothing likely to benefit the patients ever so little, too small or too low for their attention and interest. In this connexion the services of the supervisors and of those employed specially as companions to the patients, on account of the greater amount of time they may devote to it, become particularly valuable. No less important is the interest of the attendants in their various wards, nor the assistance of convalescent patients, who often confer great benefits on those around them.

"As a general rule, the evenings devoted to lectures are pretty well occupied in preparing for them, by the hour in the room, and a pleasant talk on what has been seen or heard, afterwards. The other evenings of the week should never be neglected, as there is always some danger of their being.

"In nearly all cases, life, to be really happy, must be one of action. Especially is it so in a hospital like this. From the hour of rising in the morning till that of retiring at night, except in cases of ordinary illness or high excitement, almost constant movement, change of occupation, variety of scene and surroundings, cheerful physical exercise, and prudent mental employment are needed for every day, to develope the most successful results and aid in promoting cheerfulness and tranquillity in the wards. In carrying out all these objects, it must not be forgotten that they lose half their value if done simply as a required duty, without that personal interest and hearty good-will, which rarely fails to convince patients that what is urged upon them is really intended to promote their comfort and restoration."

In all our reading of the reports of hospitals for the insane, we have met nothing more gratifying, nothing more truly indicative of a consciousness of one of the greatest stumbling-blocks of the hospital as it was, or of the necessity and the true method of removing that stumbling-block before we can obtain the hospital that is to be, than the foregoing extract. Dr. Kirkbride's words "specially tiresome," entirely fail adequately to express the character of the evenings in the hospitals of the olden time. The corridors in darkness, or each lighted by one miniature flame which only served to throw a sepulchral gloom along its wearisome length; the silence, unbroken but by the occasional raving of a maniac, or the gibberish of an imbecile; the stealthy, solemn, and solitary pacing to and fro, in the gloomiest portion of the hall, of some patient absorbed in the contemplation of his delusions; the lounging in chairs, the squatting in corners and along the side-walls, and the lying at full length, on back or on belly, upon the floor—these presented a picture—alas! *not* a picture, but a sad reality, a concrete embodiment of monotonous sluggishness, of ineffable stupidity, of a mental and physical apathy injurious to the participator, and depressing and discouraging to the beholder. As the frog, in the mathematical proposition, lost at night two of the three feet which, in his worthy endeavours to escape from the well, he had gained in the course of the preceding twelve hours, so, we have often thought, the patients subjected to, or permitted in, this course of melancholy listlessness, "fell back" in the evening through at least two-thirds of the distance toward health achieved during the day.

It needs no spirit of prophecy other than that derived from a mediocre knowledge of human nature to foretell the effects, or at least, the *tendency* of our national difficulties upon our hospitals for the insane. Those effects should, if possible, be prevented, that tendency opposed. Hence, we reproduce the following remarks, believing that they will be indorsed by every physician of long experience in the management of one of those hospitals :—

"No matter whether an institution is specially for the affluent, for the reception of all classes, or the humblest pauper hospital in the land, true economy consists in an avoidance of all waste, in having nothing done that is not useful in some way, in keeping everything in the highest state of efficiency, and doing all that is likely to restore to society its afflicted citizens in the shortest possible time. The best arrangements will always be found cheapest in the end, and the highest class of qualifications in every department, with liberal compensation, will prove more economical than inefficiency at the lowest grade of remuneration.

"In periods of unusual financial depression, when nearly every one is apt to feel the necessity for a reduction of personal expenses, there is always danger of the management of these institutions for the *cure* as well as the care of the insane, making the grave mistake of doing something as a means of lessening their expenses, that must unavoidably lower their character and impair their usefulness. Retrenchments may thus be carried to that point that they become absolute extravagance. This is clearly so if they diminish the usefulness of an institution, lessen the confidence of the public in its efficiency, and thus reduce its income to a far greater extent than they lower its expenditures. It can never be economy to neglect any available means of restoring the sick, of improving the condition of all the patients, and doing thoroughly what an institution has been specially established for. One establishment may expend scarcely more than half what another does, and yet if these expenditures are injudicious, it may be both wasteful and extravagant, while the other is truly economical."

2. Of the comparatively small, but the truly comfortable and domestic, or home-like Friends' Asylum, Dr. Worthington says:—

"Originating at a time when no institution existed in this country that could serve as a model for its plan of construction, the founders of the asylum showed, by the quantity of land purchased, and by the amount of space allotted to each patient in the construction and general plan of the building, a degree of liberality which has probably not since been surpassed. How far the asylum may have been successful in other respects in keeping pace with the progress of improvement, or how far the means employed in it, as compared with other institutions, may have contributed to the relief and restoration of the insane, is not for us to judge. It will be sufficient to say that during the past, as in previous years, we have felt the responsibility resting on us of diligently employing, for the benefit of our patients, every means within our reach which the general experience has proved to be of real utility.

"The objects of its founders, besides furnishing medical aid, and suitable moral and religious restraint, mingled with judicious kindness and sympathy, for the restoration of the insane to the inestimable gift of reason, were also to provide an asylum for the relief of those whose disease was such as to leave no hope of recovery; where they might enjoy the comforts of home so far as they were capable of appreciating them, accompanied with every liberty consistent with their welfare and safety. These objects, it is believed, have been kept steadily in view, and it has been found, by constantly increasing experience, that the amount of personal restraint on the movements of the insane, deemed necessary for their own, and the safety of others, has regularly diminished down to the present time. *We have not in any case, for several years past, considered it needful to apply mechanical restraint for the prevention of acts of violence and destruction, and we have only resorted to it in any form in cases where severe bodily disease or debility has rendered a recumbent position indispensable to the safety of the patient's life.*"

The italics are by the reviewer, and *not* by the author.

| | Men. | Women. | Total. |
|---|---|---|---|
| Patients on the 1st of March, 1861 . | — | — | 61 |
| Admitted in the course of the year . | — | — | 15 |
| Whole number . . . . . . . . . . | 32 | 44 | 76 |
| Discharged, including deaths . . . | 6 | 8 | 14 |
| Remaining, March 1st, 1862 . . . | 26 | 36 | 62 |
| Of those discharged, there were cured . | 3 | 3 | 6 |
| Died . . . . . . . . . . . . . . | 1 | 3 | 4 |

*Causes of death.*—Cerebral congestion, 1; pneumonia, 1; general paralysis, 1; chronic bronchitis, 1.

The lectures and other evening entertainments were resumed and continued through the winter. The lectures were "listened to, and the experiments witnessed with much interest, by a large proportion of the patients." From the further exposition of the plan of moral treatment we make a few extracts.

"The value of out-door occupation consists as much in the change and variety it gives from the monotony of in-door asylum life, and the mental recreation thus afforded, as in the mere physical effects of muscular exercise. Without regard to the interest that may be excited in the mind of the patient by the work he performs, there is danger that the latter, by becoming toilsome, may even prove injurious. Care is taken to avoid such a consequence by endeavouring to interest the patient in his employment, and by limiting the time spent in labour to two or three hours in the early part of each day. . . . . For those who have been unaccustomed to agricultural labour, other means of furnishing physical exercise, combined with healthy mental excitement, are resorted to. Such patients spend a portion of each day in the open air, either in walking on the premises or in the vicinity of the asylum, or are engaged in the games of quoits, cricket, or foot-ball. The latter have only been in use during the last two years, and have proved a highly valuable addition to the means formerly employed for affording exercise to this class of our patients."

"The patients are encouraged to follow their own taste and inclinations in their in-door employments. Some of them became quite expert at a particular game, such as chess or backgammon; one patient draws and paints, and has produced a number of pictures, both in oil and water-colours, which have been framed and hung on the walls. Another employed himself very successfully during the portion of the summer, in preparing the skeletons of leaves and other portions of plants; and another, who is so demented as to be almost incapable of any other employment, has become proficient in the game of battledore."

3. Our readers have already been informed of the efforts made by the managers of the Western Pennsylvania Hospital to erect, for their insane patients, a new building which should rank among the best institutions of its kind, as well as of the progress which, from year to year, they have made in their enterprise. The report now before us is embellished with a beautiful perspective view of the building as it is intended to be when complete. It is called "The Dixmont Hospital." Of its condition at the date of this report, as well as for its general plan, we quote the language of its architect, whose report is a model of compactness and perspicuity which might well be studied by scientific and literary men.

"The work on the new buildings of the Hospital for the Insane at Dixmont is now nearly completed, the plastering being finished, and the carpenters being now engaged in putting up the finishing of the wood-work inside of the building. Excepting the fixtures for heating, ventilation, and water-supply, which are all yet to be provided for, the buildings will be completed and ready for occupancy by the 1st of May next.

"The hospital proper consists of a central building, sixty-one feet front by one hundred and thirty deep, and four stories in height, arranged for the use of the officers, and to form store-rooms and chapel. On each side of this central building extends a wing building one hundred and four feet front by thirty-eight feet deep, and three stories in height, arranged with dormitories for use of the patients; each wing finishing with a building at the end of it, forty-five feet front by fifty-five feet deep, and four stories in height, arranged for day rooms of the patients. These buildings are erected with walls of brick, covered with roofs of iron, and have stone stairways, the whole being substantially constructed and finished in a neat, plain manner, the plan being arranged in reference to future extension by addition of other wings, to accommodate more patients.

"Detached from the hospital is, first, a building for the laundry, bakery, and boiler-room, forty-five feet by fifty-five, and two stories high, built of stone and covered with iron. Next, a building at the river for a pump-house, twenty-six feet square and one story high, built of stone and covered with iron roofing. Lastly, a building for station-house at the railroad, seventeen feet by thirty-two, built of brick and roofed with iron."

It is proper to state that the plan of the hospital embraces two additional wings, very similar to those already erected and to be appended to their extremities.

| | Men. | Women. | Total. |
|---|---|---|---|
| Patients in the Western Pa. Hospital, Jan. 1st, 1861 | 59 | 52 | 111 |
| Admitted in the course of the year | 63 | 32 | 95 |
| Whole number | 122 | 84 | 206 |
| Discharged, including deaths | 55 | 41 | 96 |
| Remaining, Dec. 31st, 1861 | 67 | 43 | 110 |
| Of the discharged, there were cured | 31 | 19 | 50 |
| Died | 6 | 4 | 10 |

Died of consumption, 2; exhaustion of acute mania, 2; enteritis, 2; chronic dysentery, 1; congestion of the brain, 1; typhoid fever, 1; old age, 1.

In pleading for additional facilities for moral treatment, Dr. Reed says:—

"As a relief from trouble and anxiety of mind, men resort to occupation and pleasant amusements, and when the mind has become deranged, the necessity for such treatment is greater. It is not *inaction* that is desired to accomplish a cure, but a change of *action.* In recent insanity the mind will not rest, and unless constant and urgent inducements to healthy action are presented, and new channels opened for the thoughts and affections, the patient will indulge in his perverted feelings and distorted ideas, until *dementia* places him beyond hope. It is this condition we wish to prevent, or at least postpone. We desire not to abandon the patient to blind chance, or allow him to grow worse by neglecting to provide every proper remedy."

When his patient shall have been removed to Dixmont, the doctor may look with a larger hope and a firmer faith for a more complete equipment for the battle against the disease with which he is contending.

From what follows, it appears that the insane have not lost their patriotism or their beneficence.

"One hundred and ten shirts were made for the soldiers at Washington, by the patients and employés, the materials having been purchased by the contributions of officers and employés."

4. It must have required labour to make the report of the *Bloomingdale Asylum.* What with one-third of a page of margin at its beginning, and one quarter of a page at the end, it occupies two pages.

| | Men. | Women. | Total. |
|---|---|---|---|
| Patients, Jan. 1st, 1861 | 71 | 84 | 155 |
| Admitted in course of the year | 60 | 51 | 111 |
| Whole number | 131 | 135 | 266 |
| Discharged, including deaths | 58 | 57 | 115 |
| Of the discharged, there were cured | 22 | 20 | 42 |
| Died | 12 | 7 | 19 |

"Several patients discharged improved," says Dr. Brown, "are known to have recovered after returning to their homes. The deaths were occasioned, in six cases, by acute mania, with great excitement; in two by general paralysis; in two by chronic disease of the brain with partial paralysis; in two by apoplexy; in one by suicide; in five by pulmonary consumption and marasmus; in one by disease of the kidneys. Four patients died within a fortnight after admission.

"While the average number of patients for the year as compared with that of the previous one, is scarcely less, there is an actual diminution of twenty in comparing our present household with that of some portion of last year. This falling-off is occasioned by the wide-spread pecuniary reverses, compelling the removal of many patients to State and municipal institutions."

5. The *McLean Asylum* has been enlarged by the erection of a wing intended for the cases of insanity in its more severe and violent forms. "The apartments are spacious and cheerful, and thoroughly ventilated; and for comfort, elegance, and adaptation to the use designed, are unequalled."

| | Men. | Women. | Total. |
|---|---|---|---|
| Patients, Jan. 1st, 1861 | 91 | 96 | 187 |
| Admitted in course of the year | 55 | 56 | 111 |
| Whole number | 146 | 152 | 298 |
| Discharged, including deaths | 60 | 50 | 110 |
| Remaining, Dec. 31st, 1861 | 96 | 92 | 188 |
| Of the discharged, there were cured | 31 | 23 | 54 |
| Died | 9 | 14 | 23 |

Died from chronic insanity, 8; general paralysis, 5; typhomania, 3; chronic disease of the liver, 2; phthisis, 2; epilepsy, 2; apoplexy, 1.

Dr. Tyler has devoted nearly the whole of his report to a dissertation upon the psychological condition of the country, as affected by the war.

6. The leading statistics of the report of the *Massachusetts State Lunatic Hospital*, at Northampton, are as follows:—

| | Men. | Women. | Total. |
|---|---|---|---|
| Patients, Sept. 30th, 1860 | 137 | 178 | 315 |
| Admitted in course of the year | 70 | 52 | 122 |
| Whole number | 207 | 230 | 437 |
| Discharged, including deaths | 58 | 47 | 105 |
| Remaining, Sept. 30th, 1861 | 149 | 183 | 332 |
| Died | 15 | 15 | 30 |

Died with phthisis, 9; marasmus, 8; epilepsy, 3; maniacal exhausion, 2; pneumonia, 2; cancer, softening of brain, typhomania, general paralysis, apoplexy, chronic diarrhœa, 1 each.

In allusion to the number of patients who died "from the slow wasting away which removed so many in the last stages of chronic dementia," Dr. Prince says:—

"Of these cases we have a very large proportion received from the other hospitals at the opening of this institution. This fact will for several years make our mortality larger than the average in hospitals, and also give an extraordinary proportion of deaths from chronic diseases."

It is to be regretted that the number of cures is not stated. We perceive no motive for withholding it other than that, from the proportion of chronic cases in the establishment, it is undoubtedly small. But all proper allowance would be made for that, and it is a pity that the statistics of insanity should not be kept as full and as accurate as possible.

"Our means of amusement have been enlarged by the addition of a bowling-alley, containing two boards. An appropriation of eight hundred dollars was made by the last legislature for the purpose, and a substantial brick building has been placed in a convenient spot, containing everything needed. It will soon be finished and occupied, and add greatly to the health and recreation of those for whom it was designed.

"A billiard-table was considered such an indispensable article of furniture, that one has been placed in a convenient position (thus far without cost to the institution or the State), in the hope that its importance as a means of exercise, health, and amusement, would be acknowledged, and the means of securing so desirable an article be furnished.

"Under the general head of amusements may be included the usual games made use of as relaxative, besides walking, fishing, hunting, picnics, excursions to points of interest, reading, concerts, dances, &c., all of which serve to vary the monotony of hospital life, and excite new and interesting currents of thought.

"The library has been increased somewhat, both by purchase and by donations from friends of the institution or of the patients. New pictures have been added to those which already adorned the walls. A room has been fitted as a reading-room, and supplied with the daily papers, thus supplying a want long felt and regretted."

Several pages of the report are occupied by an appeal for special provision for the treatment of habitual inebriates, but nearly the whole of it is quoted from the writings of Dr. Kirkbride.

The extract given below shows the quality of the water to the use of which the inmates of this hospital are subjected—and this, too, when "there is, upon the grounds of the institution, a never-failing spring of most excellent water, yielding a quantity sufficient, during the greater part of the year, to supply their daily wants."

"I should not feel that I had laid the matter fully before your Board if I omitted to speak of the quality of the water thus scantily and irregularly and expensively furnished. I have already, at different times, drawn your attention to the colour, and I may say the consistency of the article, as it has appeared in the reservoirs. You have found it holding in suspension so much foreign matter as to render it opaque. This matter consists of the natural debris which a rapid stream, supplied by many feeders and flowing through a soil of various composition, always carries with it. To this is added a miscellaneous mass of impurity poured into it by a great variety of manufacturing establishments. The mixture is at times disgusting to the sight and smell, and its effects equally offensive in the laundry and the kitchen. The precipitation of vegetable matter in the large boilers, as you are aware, will probably soon create a necessity for expensive repairs, by the 'burning' and 'scaling' of the iron where most exposed to the action of the fire."

7. At the *Longview Asylum*, those parts of the numerical history of the hospitals which we are accustomed regularly to reproduce, are represented by the following figures :—

| | Men. | Women. | Total. |
|---|---|---|---|
| Patients, Nov. 1st, 1860 . . . . . | 151 | ... 183 | ... 334 |
| Admitted in course of the year . . . | 111 | ... 76 | ... 187 |
| Whole number . . . . . . . . . | 262 | ... 259 | ... 521 |
| Discharged, including deaths . . . | 96 | ... 68 | ... 164 |
| Remaining, Nov. 1st, 1861 . . . . | 166 | ... 191 | ... 357 |
| Of the discharged, there were cured . | 67 | ... 48 | ... 115 |
| Died . . . . . . . . . . . . . | 12 | ... 14 | ... 26 |

Died from phthisis, 10; general paralysis, 5; maniacal exhaustion, 4; senile debility, 2; apoplexy, 2; epilepsy, 1; marasmus, 1; cerebral congestion, 1.

"Quite a large number died of diseases not directly connected with their insanity. Many of them were very old patients; half of them had been in asylums more than ten years; some were inmates of the lunatic department of the Commercial Hospital, Cincinnati, as far back as 1832, beyond which there are no records of that institution.

"There has been but little sickness in the house originating after the admission of the patients; and there has been entire freedom from epidemics, or diseases of any kind depending upon general causes, such as location, &c. In one respect this has been quite remarkable, although something similar has been observed in other asylums. Intermittent fever has prevailed extensively in the immediate neighbourhood of the institution, and has attacked several of the residents, including the superintendent; but not one of the insane has been at all affected by it.

"Of the whole number of recoveries, ninety-two were of patients admitted during the year; and much the larger portion were brought to the asylum within a month of the commencement of the disease. Of those who had been labouring under the disease one year or more before being brought in, but little over one-fourth recovered."

While treating of the origin of insanity, Dr. Langdon, judging from his own patients as well as from the opinions of other physicians, says: "By far the greater number of cases are produced by causes which directly depress the vital energies. This is farther shown by the condition of the great majority, almost all, of the patients when they came into the house. Feeble, depressed, and emaciated, they require tonics and supporting treatment, and generally show improvement in the state of their minds as their physical strength improves."

As the Longview Asylum is intended chiefly for paupers, and as the old establishment, for which it is the substitute, was but poorly provided with the means of moral treatment, we rejoice in the assertion that "during the year there have been many additions to our means of recreation and amusement. Among these are a bowling-alley, a magic lantern, a melodeon, singing-birds, flowers, and pictures."

The annexed paragraphs are worth reading, both for their further exposition of the moral treatment, and for the discordance of some of the opinions therein expressed with the opinions of some others of the superintendents of hospitals:—

"Once in each week we have had dancing parties, which have been attended by a large number of the patients with evident benefit, in some cases increasing their cheerfulness, exciting more interest in external matters, withdrawing them from the contemplation of their morbid fancies, and stimulating their minds to activity in a new direction. These parties are very much enjoyed, and anticipated with great pleasure by all the patients who are able to attend them; furnish material for conversation both before and after, and seem to be generally productive of good. The same may be said of concerts, which we occasionally have of an informal kind—music, both instrumental and vocal, having a quieting effect at the same time that it tends to break up their habitual morbid train of thought.

"Religious services have been discontinued for a long time, as they do not appear to have any beneficial effect, and sometimes do positive harm. The religious education and belief of the patients differs so widely that it is almost impossible so to arrange it as not to offend and excite some portion of them when any religious services are held. We have Jews and Christians,

Protestants and Catholics, Spiritualists and Infidels, in the house, all as firmly convinced of the truth of their own particular belief and as intolerant of any other as if they were perfectly rational, and any appearance of favouring one or the other form excites prejudice, and destroys that trust and confidence which is of so much benefit in the treatment of the insane.

"It can hardly be claimed that there is a necessity for religious exercises among persons not able to transact the ordinary business of life in consequence of their disorder of mind. Nor would it be easy to find a person so peculiarly gifted as to be able to conduct such exercises in a manner that would at least do no harm.

"For reasons somewhat similar, no funeral services are had at the institution; the mere knowledge of a death taking place is of itself depressing, and has a pernicious influence on many of the inmates, and to impress it on their minds by funeral services seems to me would be productive of only bad results."

Of the 521 patients treated in the course of the year, only 163 were natives of the American continent, while 353 were Europeans.

8. The general items of the register of the *New Hampshire Asylum*, for the year terminating with the close of April, 1862, are as follows:—

| | Men. | Women. | Total. |
|---|---|---|---|
| Patients at the beginning of the year . | 88 | ... 108 | ... 196 |
| Admitted in course of the year . . . | 45 | ... 41 | ... 86 |
| Whole number . . . . . . . . . . | 133 | ... 149 | ... 282 |
| Discharged, including deaths . . . | 45 | ... 49 | ... 94 |
| Remaining at the end of the year . . | 88 | ... 100 | ... 188 |
| Of the discharged, there were cured . | 20 | ... 21 | ... 41 |
| Died . . . . . . . . . . . . . | 10 | ... 3 | ... 13 |

Died with epilepsy, 5; "dropsy from disease of heart," 1; "disease of the heart originating in rheumatism," 1; chronic pleurisy, 1; exhaustion of acute mania, 1; general paralysis, 1; organic disease of the brain, 1; exhaustion of chronic insanity, 2.

"Much sewing, knitting, and similar work has been done by the female patients, which has a second value, great, though less than that accruing to the labourer herself. This is emphatically true of a considerable amount of sewing and knitting done for the army. No occupation has seemed to afford them more unalloyed pleasure than that done for the noble and self-sacrificing defenders of our country."

The hospital having completed the twentieth year of its operations, the occasion is seized by Dr. Bancroft to give a brief sketch of its history, with reflections suggested by its results. From the account of the proceedings preliminary to the action of the Legislature authorizing the construction of the hospital, we take the subjoined extract:—

"At length a call was made on the several towns for statistical information as to the number and condition of the insane, and the responses to this call early confirmed their previous convictions. Returns were received from 161 towns. Of these only 20 were without insane persons. In the balance of the towns there were 312 insane, and of these 152 were entirely a public charge, 160 being independent of public charity. Of the whole number, 81, or nearly 26 per cent., were confined in cages, jails, close rooms, handcuffs, or the like. Insanity had existed in these individuals for periods varying from a few weeks to sixty years, the average of the whole being about thirteen and one-half years. In the report of the committee deputed to collect these facts, made to the Legislature in 1836, they declare 'that the horrors of the present condition of the insane are far from having been exaggerated.'"

The following selections are from the remaining portion of this section of the report:—

"The theory and policy of the guardians, as well as the successive physicians, has been to reduce the difference between the wards of the Asylum and a well-regulated household, to the lowest point consistent with safety, and the integrity of the best curative agencies."

"Even in the wreck of reason and responsibility, nature has kindly provided that the human being need not be wholly a wreck. Such is the organization that, like an automatic machine, when the intelligent, directing mind is cast from its supremacy, still, under a law of habit, if the individual can be under the influence of another mind to keep him in motion, he will follow to a great extent the routine of ordinary life, when, without this exterior influence, he would subside into stupid inaction, and fall under the direction of the mere animal instincts. It is by availing themselves of this principle, that asylums, in cases of the incurable, are able to transform what would otherwise be little more than existence into a life possessing many rational occupations and enjoyments."

"Of the 1927 who have been received into the Asylum, 841 have been restored to mental soundness. . . . . It is not easy to calculate the value of the restoration to society of this considerable number of insane, or to estimate the amount of pain and sorrow mitigated or relieved by their escape from a fate more to be dreaded than death. But whatever society, the domestic circle, or bleeding hearts, may realize from these restorations, it is of small account compared with the reinstating of reason in the mind from which it had been dethroned; the restoring of delicate sentiments and affections, the perversion of which had changed the sweets of life to bitterness."

The first superintendent of this institution was Dr. George Chandler, afterwards superintendent of the Massachusetts State Hospital, at Worcester, and now a resident of that city, but retired from active duty in the profession. His successors have been Dr. Andrew McFarland, now having charge of the State Hospital of Illinois, Dr. John E. Tyler, now at the head of the McLean Asylum, and Dr. J. P. Bancroft, the present incumbent.*

---

### Art. IX.—HYPOCHONDRIASIS.

* By Dr. Pliny Earle, from the *American Journal of Medical Science*, for January.

